# Social Responsibility and Urban Consolidation Centres in Sustainable Freight Transport Markets

**DOI:** 10.1007/s40797-022-00204-4

**Published:** 2022-07-22

**Authors:** Daniele Crotti, Elena Maggi

**Affiliations:** Department of Economics, University of Insubria, Via Monte Generoso, 71, 21100 Varèse, Italy

**Keywords:** Corporate social responsibility, City logistics, Urban distribution centres, Logistics service providers, Hotelling competition, D43, M14, Q53, R41

## Abstract

This paper investigates the impact of Corporate Social Responsibility (CSR) strategies adopted by urban consolidation centres on the competition between logistics service providers. Taking into consideration pollution charges issued by city councils to reduce CO_2_ emissions in freight distribution, we study a Hotelling-like market setting where logistics providers could voluntarily outsource last mile deliveries to platforms endowed with eco-friendly vehicles. We considered the intensity of the competition, which is captured by switching costs and we theoretically find that in more contestable markets, the interplay between environmental policies and CSR strategies—intended as consumers-oriented pricing schemes—is more likely to enhance the demand by providers for consolidation centres’ deliveries. Conversely, when the competition is weak, i.e., the providers have a relatively strong market power, platforms’ services are less attractive and more relevant CSR pricing strategies are needed to spread out greener freight deliveries and make city logistics environmentally sustainable.

## Introduction

**Fig. 1:**
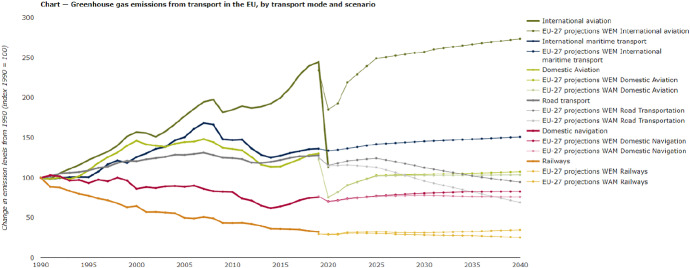
Projections of transport-related emissions (EEA 2020) (https://www.eea.europa.eu/data-and-maps/indicators/transport-emissions-of-greenhouse-gases-7/assessment-2)

Urban freight transport (UFT) plays a crucial role in the achievement of Sustainable Development Goals (SDGs), especially those related to climate action and sustainable cities and communities (United Nations 2015). The growth of e-commerce and B2C sales (intensified with the Covid-19 pandemic) has dramatically expanded freight urban transport flows, increasing congestion level and worsening air quality in the cities (Sarkis 2020; Kohli et al. 2020; Holguín-Veras et al. 2016; McKinnon 2010). In Europe, road transportation indeed causes the highest rate of overall transport emissions: around 72% in 2019 of all domestic and international transport GHG (EEA 2020). According to the estimates, after the drastic fall of emissions in 2020, due to the reduction of transport activities during the initial spread of Covid-19 pandemic, we would expect a significant rebound in transport emissions by 2040 compared with 1990 levels (EEA 2020; see Fig. 1, grey line). However, only the application by the European Member States of additional measures to reduce transport emissions could help in reducing them after 2022. Since the majority of planned or implemented measures regards road transport, the share of this mode is expected to decrease faster than other transport modes, but this process is strictly related to the level of effectiveness of the sustainable transport policies that are now concentrated more on passenger transport than on the freight one. As a result, the sprawling of logistics hubs and inland terminals in the nearby of or within the cities, namely Urban Consolidation Centres (UCCs), aimed at increasing the efficiency and sustainability of transport systems covering last-mile markets, remains a crucial issue (Vidal Vieira and Fransoo 2015; Dablanc and Rakotonarivo 2010; Dablanc 2007).

Since a mix of environmental policies has been widely advocated to reduce the road traffic pollution, according to the polluter-pays principle (EC 2004) several municipalities have widely imposed market-based restrictions to limit the number of polluting vehicles and to let UFT operators internalize negative externalities on the air quality (Müller and Petit 2019; Lebeau et al. 2017). Along with designing pollution charges to enter Low Emission Zones (LEZs) or similar measures (De Marco et al. 2017; Danielis et al. 2013), several city councils tested Urban Consolidation Centres, i.e., facilities located in the proximity of a city centre, which collect last-mile deliveries outsourced by international or national LSPs and distribute them to the final urban destinations with fully loaded small vehicles, which are normally electric or of low emissions (Cleophas et al. 2019; Holguín-Veras et al. 2015; Crainic et al. 2009). According to the most quoted literature, UCCs are considered generally effective in reducing CO_2_ and NO_2_ emissions (Katsela et al. 2021; Holguín-Veras et al. 2015), but the long-term survival of those platforms is harmed by some management and financial problems: an unstable demand from LSPs in the long run and *double marginalization* (Marcucci and Danielis 2008; Browne et al. 2007). Regarding the first point, branded LSPs do not have the right incentives to outsource deliveries (Holguín-Veras and Sánchez-Díaz 2016; Soh et al. 2015), also because thus far the freight transport providers had a weak orientation towards CSR-based pricing schemes. Actually, even though these strategies are different for each industry (Sweeney and Coughan 2008; Ciliberti et al. 2008), in the supply chain and logistics sector they consist in: (1) the size and complexity of transportation networks; (2) the necessity to provide customers with personalized services; and (3) the need of cooperative solutions with other industry players and local stakeholders involved in the supply chain, including actions to look after the whole consumers’ welfare (Isa et al. 2021; Gatta et al. 2019; Paddeu 2018; Marchet et al. 2014). Concerning the second problem, adding up a distribution layer to existing supply chains tends to raise final freight transport rates, thus reducing the cost efficiency of whole parcels distribution (Janjevic and Ndiaye 2017). In this paper, we consider how the enhancement of CSR-oriented policies might make the UCCs more attractive for the LSPs. Generally intended as pricing strategies increasing the utility of all the final consumers of goods and/or services (Porter and Kramer 2011), socially responsible and stakeholders-related policies in the UFT markets recently emerged as of key importance, also due to market signals coming from more *environmentally conscious* consumers (Björklund and Johansson 2018).

Following a recent and growing literature about the strategic role of CSR in oligopolistic competition (e.g., Planer-Friedrich and Sahm 2020; Kim et al. 2019; Hirose et al. 2017), the paper addresses the following questions: (1) what is the UCC impact on the competition among LSPs in the UFT markets?; (2) would CSR pricing schemes increase the LSPs’ demand for UCCs?; (3) considering UCCs, is there a competition interplay between environmental policies and CSR pricing schemes?

After reviewing some successful UCC experiences considering CSR practices, we put forward the idea that the application of those strategies could enforce the public environmental policies, by giving a contribution to the challenge of fighting the climate changes in urban areas, decarbonising freight transport market. Consequently, UCCs could become a long-term winning solution for UFT sustainability (Lagorio et al. 2016). Moreover, referring to competition, CSR practises by UCCs could answer to the need of LSPs of increasing their environmental commitment in the light of their customers. Therefore, UCCs could stabilize their own demand and become more attractive than in the past (Björklund et al. 2017). To the best of our knowledge, this is the first study considering the interplay between CSR strategies and the oligopolistic competition among LSPs in last mile freight transport market. We designed an Hotelling-like model of UFT competition, based on the assumption that CSR-oriented firms maximize a combination of profits and consumers’ welfare (Planer-Friedrich and Sahm 2018; Königstein and Müller 2001).

The paper is organized as follows. In Sect. 2, a background on CSR in logistics and strategic competition is provided, including a qualitative review on CSR strategies in UCC cases. A model to study the impact of CSR strategies by UCCs on the competition among LSPs is presented in Sect. 3, while Sect. 4 concludes the paper with final remarks.

## Background and Literature Review

Our paper gives a contribution to three different strands of literature, as follows.

### CSR-Based Pricing Schemes in the Logistics Sector

**Fig. 2:**
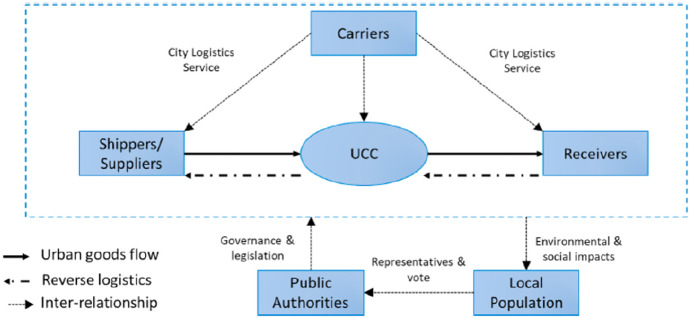
Network of UCC’s stakeholders (Isa et al. 2021)

Even though, in the last decades, the adoption of socially responsible strategies has become a global practice also in the logistics industry (see, e.g., Piecyk and Björklund 2015; Ciliberti et al. 2008; Carter and Jennings 2002), this issue has been not yet largely explored by the literature. In particular, very low attention has been given to the impact of CSR on the city logistics, where the transport operators’ cross-functional nature can promote policies aimed at ensuring environmental and social sustainability (Isa et al. 2021; Oberhofer and Dieplinger 2014). The communication of social and environmental engagement to receivers and the public audience, by reporting CSR activities, indeed can be crucial for LSPs competition in the market (Uyar et al. 2020; Tate et al. 2010; Porter and Kramer 2011). However, it is also recognized that LSPs face hard challenges when trying to carry on their own sustainability agenda, due to the complexity of network-based actions, the design of receivers-led strategies, and above all the need of cooperation between private/public stakeholders (Gatta et al. 2019; Holguín-Veras and Sánchez-Díaz 2016; Abbasi and Nilsson 2016). In fact, past studies have highlighted the high complexity of the city logistics market, where many different stakeholders, i.e., LSPs, goods’ receivers, public institutions and policymakers interact (Carvalho et al. 2019b; Geßner et al. 2013; Gold et al. 2010; Taniguchi et al. 2001; Maggi 2007). The Fig. 2 shows this complexity and the different actors involved, including the UCC, which positions itself in the logistics chain as an intermediary between the shippers and receivers, delivering the goods in the last mile or colleting reverse logistics components on behalf of different carriers (LSP) in urban areas. Public authority and local population are the stakeholders indirectly involved in city logistics process. The first has the task to regulate urban transport and its related level of pollution, pursuing the general interests of the community. The second covers the triple role of consumers (final goods receivers, especially in e-commerce transactions), of citizens voting local government (also considering its ability to guarantee a high quality of life in the city) and finally of third party affected by transport environmental and social impacts.

Despite the advocated importance of corporate social and environmental policies, there are also relevant reasons why CSR-based pricing schemes are not easily adopted by LSPs. From a competition perspective, potential drawbacks might be detected for both low and high differentiation between providers. In general, it has been recognized that the relationship between CSR strategies and stakeholders’ interests has a striking impact on LSPs’ differentiation strategies and related market shares (Uyar et al. 2020; Johansson and Björklund 2017). On the one hand, when the competition is relatively fierce (i.e., providers’ market power is not large), the long-term renewal of vehicle fleet and related investments in green technologies by LSPs are often made complex because financial resources are generally lacking (van Heeswijk et al. 2020; Marchet et al. 2014; McKinnon 2003). On the other hand, when incumbent logistics companies are highly differentiated, their market power does not give the right incentives to innovate own technologies, unless an increasing consumer demand for ‘green’ products or the existence of restrictive public environmental policies (Katsela and Pålsson 2020; Björklund et al. 2016). As a result, this implies that LSPs might have a hard time in ‘making’ and sustaining in-house CSR-based pricing schemes (Piecyk and Björklund 2015).

### Sustainable Deliveries and UCCs

The second strand of literature to which we refer deals with one of the most promising solutions for LSPs to comply with environmental-friendly requirements, i.e., outsourcing last mile consignments to UCCs (Ciardiello et al. 2021; Panero et al. 2011; Brown et al. 2007). However, although UCCs can reduce CO_2_ emissions in UFT markets, a crucial drawback could limit their ability to increase and/or stabilize their demand from LSPs: the addition of one layer to supply chains, from a vertical perspective, potentially implying higher costs for LSPs (Deng et al. 2020; Holguín-Veras et al. 2016; Nordtømme et al. 2015; Van Duin et al. 2012; Quak and Tavasszy 2011).

Several attempts to establish successful UCCs have been made in the last 30 years (Allen et al. 2012). According to recent reviews (e.g., Trentini et al. 2015; Allen et al. 2014), 25 UCC projects in the UK, 14 in Germany, Italy and the Netherlands, and 11 in France have been undertaken. In France, the UK and the Netherlands about 33% of UCCs went beyond feasibility studies, while in Germany and Italy about 40% have become fully operational. Apart from different success rates at a country level, Björklund et al. (2017) identified few factors influencing the viability of UCCs, among which one aspect emerges as being crucial: the creation of value for society and citizens (including the explicit mission for environmental goals). However, as synthetized in the Table 1, the application of CSR-based pricing schemes by UCCs themselves (relying upon the consideration of overall consumers in the market) has not been detected in many initiatives, especially those who turned out to be not attractive for LSPs (Van Duin et al. 2010; Marcucci and Danielis 2008) and not financially viable in the long run (Carvalho et al. 2019a; Janjevic and Ndiaye 2017). By contrast, some key examples of successful UCCs have displayed a relevant care for all the final customers (or parcels receivers) in the UFT market, by combining profit-maximizing and welfare-enhancing goals in their own pricing schemes.

**Table 1:** Review of selected UCC projects and related CSR-based pricing schemes

Location	UCC project	Duration	CSR strategies (receivers’ welfare)	References
La Rochelle (France)	ELCIDIS	2001 (pending)	Involvement of commercial sectors and retailers	Patier (2006)
Leiden (Netherlands)	Leiden UCC	1994–2000	Not developed	Schoemaker et al. (2006)
Kassel (Germany)	City Logistik	1994–2005	Not developed	Van Duin et al. (2010), Köhler (2004)
Bristol – Bath (UK)	BBFCC	2004—to date	Retailers-based pricing	Paddeu (2018), Allen et al. (2014)
Padua (Italy)	CityPorto	2004—to date	Pricing schemes shared with local council	Björklund and Johansson (2018), Janjevic and Ndiaye (2017), Gonzalez-Feliu and Morana (2010)
Lucca (Italy)	Lucca Port	2007 (pending)	Shared responsibility with private stakeholders	LIFE ASPIRE project, 2021 (http://www.life-aspire.eu/), Björklund et al. (2017)
Parma (Italy)	Ecocity	2008—to date	Strong connection with municipality and customers’ associations	Morganti and Gonzalez-Feliu (2015)
Barcelona (Spain)	SuperBlock	2016 (pending)	Not developed	Brass (2017), Hu (2016)
Gothenburg (Sweden)	Stadsleveransen	2013—to date	Inclusion of whole market interests (e.g., trade unions)	Björklund et al. (2017)
Huddinge (Sweden)	Södertörn 8	2014—to date	Pricing schemes in line with whole customers’ interests	Genovese et al. (2017)

Starting from selected UCC projects that are not active anymore, they all displayed a weak or not enduring attention to the integration of whole society and stakeholders’ interests in their business, that has been instead recognized as an aspect more important than logistics issues themselves (Björklund and Johansson 2018). An example is provided by the UCC in Leiden (Netherlands) started in 1997 as a public–private partnership (Schoemaker et al. 2006). The objective was to deliver 500 shipments per week to the city centre, but only about 25 retailers in the city centre were supplied by the UCC. The Leiden project was stopped in 2000 due to the shortage of participating LSPs, whose receivers-led needs were not inherently met (Johansson and Björklund 2017). Similarly, as argued by Van Duin et al. (2010), the 2004 UCC project in Kassel (Germany) suffered from a collapse in revenues due to stopped subsidies in 2008, but also because of the lack of strategies targeted to receivers (see also Köhler 2004). More recently, within the framework of the local “Urban Mobility Plan”, the city of Barcelona started in 2016 the ‘Superblock’ initiative (Hu 2016). Although positive results in terms of air pollution and freight delivery have been achieved, this case fairly illustrates the impact of inadequate stakeholders-oriented policies. Still active, yet the project struggles to get a stable turnover, due to its weak acceptance by LSPs, a lack of long-term budgeting, and receivers’ scepticism (Brass 2017).

Turning to successful examples of UCCs, many of these have displayed in the last years a clear attention to stakeholders’ perspective, especially by applying pricing schemes shifting from only profit-maximization perspective (Van Duin et al. 2010; Kohli and Jaworski 1990) to a society-oriented commitment (Planer-Friedrich and Sahm 2018). In case of last-mile freight transports, all industry customers’ interests are essentially incorporated in tailored pricing schemes, which enclose the welfare of the market retailers, public bodies, and shopkeepers (Österle et al. 2015). In 2007, Bristol was involved in the CIVITAS-RENAISSANCE project, aimed at developing a UCC also in the city of Bath (Allen et al. 2014). The municipality of Bristol (BCC) provided early funds to finance a UCC supplying the retailers of both the cities, at the same time imposing traffic and access restrictions to incentivize the retailers to join the scheme. Yet, since no other costs were covered by the BCC, the UCC managers set reduced service fees, encouraging retailers to patronize LSPs using UCC services. The retailers participating to the scheme were 81 in Bristol and 25 in Bath (Paddeu 2018). At date, the Bristol-Bath Freight Consolidation Centre (BBFCC) is one of the most successful schemes. Similarly, La Rochelle UCC, initiated in France in 2001, benefited from the subsidies initially provided by the local government and from municipality policies aimed to encourage LSPs to drop off their goods at the UCC. According to Patier (2006), the success of the UCC is primarily due to the awareness of all stakeholders, especially urban commerce, shared with local administrators and policy makers, of the importance of acting quickly to reduce air pollution. Despite that, in the last years the project survival has been in danger, and from 2016 the La Rochelle Urban Community adhered to the URBACT project to strengthen the UCC strategies on the stakeholders’ side. This permitted an in-depth assessment of the current situation and the decision to relaunch the UCC in La Rochelle (https://urbact.eu/).

Established in 2008, the “Agri-Food & Logistics Centre—CAL” was designed as an Urban Consolidation Centre platform in order to manage last mile deliveries of agri-food goods within Parma’s city centre. The project was first financed by the Emilia-Romagna region and the local municipality, which created “EcoCity”, a company owned by Parma’s city council that also managed all its operations (Morganti and Gonzalez-Feliu 2015). Although seemingly very good from an operational and environmental point of view, the EcoCity project first encountered similar issues as many city logistics pilot projects faced before it. However, the ability of UCC’s manager to involve the municipality and various consumers’ association let the project stay alive and temporarily overcome financial distress.

The Södertörn UCC initiative is focused on consolidating supplies to public schools within Huddinge, in the Stockholm sub-region. A total of 8 municipalities participated in the scheme, which transited into an operation in 2017, following 2 years of trial. Some emergent indicators of its successful transition—documented in the Prosfet project (http://www.prosfet.eu/)—include early and pre-implementation consultation with a wide range of stakeholders, including the consideration of whole customers’ interests prior to the commencement of the project phase (Genovese et al. 2017).

Opened in 2007, the Lucca Port project is an UCC managed and owned by the city council of Lucca in Tuscany, Italy (Björklund et al. 2017). The project has been running for several years without generating the expected results, causing the substitution of the initial management firm with a new one in 2015. However, since this strategy has not been successful, the service stopped in 2018. By getting funded by the Life Aspire project, a European initiative aimed at promoting sustainable urban logistics, the city council is now trying to renew the project, especially by promoting a shared responsibility among commercial stakeholders, collaborating with them, and promoting sustainable freight deliveries in the Lucca’s city centre (http://www.life-aspire.eu/). Still referring to Italy, it worth to mention the CityPorto project in Padua, which represents the urban logistics strategy implemented by the local city council in 2004 to manage last mile deliveries within the city centre, utilising low-emission and electric vehicles (Janjevic and Ndiaye 2017). From the beginning UCC strategies included the decision to collaborate with incumbent LSPs, and the service performance has been outstandingly improving over time (+ 126% deliveries between 2005 and 2015). The main strength of this project has always been the establishment of a win–win dialogue between the UCC’s management, urban receivers, local LSPs and the city council, particularly interested in enhancing the welfare of the entire UFT market (Björklund and Johansson 2018; Gonzalez-Feliu and Morana 2010). Following the successfully example of the CityPorto in Padua, in the very last years some Swedish projects reinforced the importance of considering not only *own* final customers’ welfare but also the city receivers’ utility as a whole. For instance, being managed by interacting organisations (including the municipality and a trade organisation representing the city’s retailers), the UCC in Gothenburg aimed at making more sustainable city logistics; in 2017, it failed to break-even, becoming financially viable, for only 15% (Björklund et al. 2017).

### CSR-Oriented Behaviour by Oligopolistic Firms

Lastly, from a methodological perspective, this paper gives a contribution to the growing theoretical literature on the strategic choice of private firms. Overall, the traditional economics distinguishes between the shareholder theory, postulating that firms should look after investors’ interests, and the stakeholder theory, under which other individuals and/or social groups affected by companies’ actions should be protected by regulation, law and contracts (Benabou and Tirole 2010). From the former viewpoint, CSR strategies could be included among firms’ variables, reflecting management boards’ ways to engage in society-oriented goals (or even philanthropy) to strengthen their market position and/or align own interests with those of public institutions (Kemper et al. 2013; Kitzmueller and Shimshack 2012; Königstein and Müller 2001). About the strategic motivations for adopting CSR behaviours, in the last years, several scholars formulated models to study how the adoption of CSR schemes might impact on market settings. Clearly, since this approach would have positive effects for the society only if marketed goods and/or services (and the production technologies involved) can really improve social and/or environmental conditions, it happens that apparent (or not effective) CSR actions may give firms higher profits, but any benefit to the society (e.g., see Hirose et al. 2017; Brand and Grothe 2015; Liu et al. 2015). From a stakeholders-based view, instead, it was also recognized that CSR is a corporate governance tool, as firms have to meet the normative goals established from the social contract (Kim et al. 2019; Kohli and Jaworski 1990). In other words, a modern company should channel the expression of society’s values, and management boards could be inherently committed to sacrifice parts of profits for a good cause (on this point, see also Benabou and Tirole 2010). Following this approach, in this paper we model UCCs as facilities where managers could (voluntarily) go beyond contractual obligations and embrace pricing schemes, considering the society’s welfare as a strategic target. Essentially, a typical feature shared by that literature is the assumption that CSR actions aim at maximizing an objective function given by a weighted sum of profit and the whole consumer surplus. Thus, from the UCC’s perspective, CSR incentives consider the welfare of all the customers in the market (Planer-Friedrich and Sahm 2020).

## Impact of CSR Strategies of UCCs on the Competition Among LSPs

Once considered the importance of stakeholders’ interest involvement for the success of a UCC, we aim to answer to our main research question by studying the impact of UCCs and their possible CSR strategies on the competition among LSPs. By developing a simple duopolistic UFT market model, we contrast two sequential scenarios, one where the UCC is not active yet and pollution charges are the only measures to reduce transport-related CO_2_ emissions; the other one where an UCC is in operation, and its managers may adopt CSR-based pricing schemes.

### Benchmark (Pre-UCC) Scenario

We start considering a city logistics market, where the demand for last-mile deliveries is assumed to be met by two LSPs (A and B), which offer one freight transport service for each customer. Without loss of significance, the LSPs are modelled as they are similar in terms of technology, cost structure, and size, except for their location along a unit linear city centre (e.g., A located at 0 and B at 1). City receivers (mostly retailers and shopkeepers whose interests are commonly looked after by municipalities) are uniformly distributed along the market line and are assumed to be willing to pay higher freight transport rates to a certain LSP if switching costs to change the provider are enough high (implying a relatively strong customers’ loyalty). In case of oligopolistic logistics operators, this hypothesis is rather standard, as the long-term loyalty in the freight industry is often guaranteed by search costs (Dell’Olio et al. 2017). As a result, if LSPs post identical rates, receivers with a low (high) *x* patronize A (B) over B (A), according to a horizontal differentiation based on the willingness to adopt different providers (Soh et al. 2015; Yang and Peterson 2004; Daugherty et al. 1996). Following the Hotelling tradition (Anderson and Wilson 2015; Hotelling 1929), thus receivers enjoy a net utility when buying one unit of delivery services as follows: $V_{A} \left( x \right) = v - tx - f_{A}$ if patronizing A, and $V_{B} \left( x \right) = v - t\left( {1 - x} \right) - f_{B}$ if patronizing B. Not patronizing any LSP (i.e., relying on own-account deliveries) is assumed to convey negative utility. As we are modelling receivers belonging to commercial sector, we consider them as being cost minimizers, thus (for sake of simplicity) their willingness to pay for deliveries $v$ is symmetrical and normalized to 1. In a sense, a fixed reservation price means that receivers (i.e., retailers, shopkeepers, etc.) must reduce input costs to be competitive in their own selling market. Freight transport rates are labelled as $f_{A}$ and $f_{B}$, respectively, while, according to traditional Hotelling-like models, switching costs (or the level of receivers’ loyalty to incumbent LSPs) are measured by *t* > 0. From the supply side, the operating costs (e.g., vehicles maintenance, handling, tracing and tracking, etc.) could be set to zero (including fixed costs that do not influence freight rates in the short-run competition). Without loss of generality, we take into consideration the impact of local environmental policies on LSPs, by also assuming pollution charges set by municipalities to help reducing environmental nuisances and CO_2_ emissions. Hence, when entering LEZs, A and B must pay (symmetrical and marginal) pollution charges $c>0$.^2^

Along the linear city centre, a location exists such that a receiver is indifferent between patronizing A or B. That location is given by equating the net utilities, i.e., $V_{A} \left( x \right) = V_{B} \left( x \right)$, and the LSPs’ demand functions are:

1 $$ D_{A} \left( {f_{A} ,f_{B} ;t} \right) = \left( {t + f_{B} - f_{A} } \right)/2t\;\;{\text{and}}\;\;D_{B} \left( {f_{B} ,f_{A} ;t} \right) \triangleq 1 - D_{A} \left( {f_{A} ,f_{B} ;t} \right) = \left( {t + f_{A} - f_{B} } \right)/2t $$

Considering demands in (1), the LSPs’ profits, $\pi_{i} \left( {f_{i} ,f_{j} ;t,c} \right) = \left( {f_{i} - c} \right)D_{i} \left( {f_{i} ,f_{j} ;t} \right)$, for $i,j = A,B$, are maximized by setting respective freight transport rates, i.e., $\partial \pi_{A} \left( \cdot \right)/\partial f_{A} = 0$ and $\partial \pi_{B} \left( \cdot \right)/\partial f_{B} = 0$. By solving the resulting system, as in localized models, we derive symmetrical equilibrium rates, $f_{A} \left( {t,c} \right) = f_{B} \left( {t,c} \right) = t + c$, entailing the degree of horizontal differentiation between LSPs (based on switching costs/customers’ loyalty) and pollution charges.^3^

Yet, as we are modelling an UFT setting where the market is covered (i.e., LSPs’ market shares are not harmed by own-account deliveries performed by receivers), a further qualification of pollution charges is at point here. Actually, for a UFT market being fully covered by the two LSPs, all the receivers must gain a positive utility being served by A or B, that is, for $c \le 1 - \frac{3}{2}t \equiv \overline{c}\left( t \right)$.^4^ As a result, since pollution charges are channelled to final rates (thus lowering receivers’ utility), we let them be set at their upper-bound level for the UFT market being fully covered, i.e., $c = \overline{c}\left( t \right)$. Notice that, for $\overline{c}\left( t \right)$ to be positive, it must be $t < \frac{2}{3}$.

#### Remark 1

*When the horizontal differentiation among LSPs is strong (weak), providing high (low) switching costs between LSPs, the UFT market is fully covered for relatively lower (higher) pollution charges.*

This feature lets the model come close to real-life UFT markets, where the bargaining power between LSPs and receivers is generally skewed towards the latter; consequently, the receivers have the upper hand in the pricing negotiation and, on average, they decide to contract LSPs if generalized costs are not too high (for this point, see Holguín-Veras and Sánchez-Díaz 2016).

By imposing the above condition on pollution charges, after some algebra we back up the pre-UCC equilibrium freight transport rates, market shares and profits:

2 $$ f_{A} \left( t \right) = f_{B} \left( t \right) = 1 - t/2;D_{A} \left( t \right) = D_{B} \left( t \right) = 1/2\;{\text{and}}\;\pi_{A} \left( t \right) = \pi_{A} \left( t \right) = t/2 $$

Finally, by defining the social welfare *SW* as the sum of LSPs’ profits and consumer surplus, in the pre-UCC scenario we get:

3 $$ SW\left( t \right) = \pi_{A} \left( t \right) + \pi_{B} \left( t \right) + \mathop \int \limits_{0}^{{D_{A} }} \left( {1 - tx - f_{A} } \right)dx + \mathop \int \limits_{{D_{A} }}^{1} \left( {1 - t\left( {1 - x} \right) - f_{B} } \right)dx = 5t/4. $$

### Post-UCC Scenario

Now we consider a market scenario in which local environmental policies are paired with delivery services supplied by an UCC, who aims to be also relatively consumer-friendly. Thus, the managers would act by maximising a weighted sum of own profits and the whole consumer surplus, according to a CSR-oriented mission (e.g., Kim et al. 2019), as explained in Sect. 2. In this case, if one of the two LSPs (say, B) voluntarily outsources *last-mile* deliveries to the UCC,^5^ from a pricing perspective, a downstream stage is added to its supply chain. We refer to UCC service fees (including handling, drafting, etc.) that raise final freight transport rates, due to the *double marginalization* effect (e.g., Janjevic and Ndiaye 2017; Marcucci and Danielis 2008). Actually, in the UFT markets it is widely recognized that final customers do not wish to change the goods’ procurement modality, especially if such change involves increased costs, either in terms of time or money (Dell’Olio et al. 2017). Since an operator is added to the UFT supply chain, the timing of the market interaction is extended, allowing for two stages. In the first stage, the UCC sets service fees, depending on its own CSR strategies. In the second stage, given UCC fees, the LSPs simultaneously set freight transport rates by considering their demand. In order to derive the subgame perfect Nash equilibrium (SPNE) of the post-UCC market game, we proceed backward, starting from the second stage. As a result, in the post-UCC scenario (labelled with *U*), the receivers’ utility functions are $V_{A}^{U} \left( x \right) = 1 - tx - f_{A}^{U}$ if being served by A (who does not apply for UCC services), and $V_{B}^{U} \left( x \right) = 1 - t\left( {1 - x} \right) - f_{B}^{U}$ if being served by B (who outsources deliveries to the UCC). Again, equating the utilities, the demand functions become:

4 $$ D_{A}^{U} \left( {f_{A}^{U} ,f_{B}^{U} ;t} \right) \!=\! \left( {t + f_{B}^{U} \!-\! f_{A}^{U} } \right)/2t\;\;{\text{and}}\;\;D_{B}^{U} \left( {f_{B}^{U} ,f_{A}^{U} ;t} \right) \!=\! \left( {t + f_{A}^{U} - f_{B}^{U} } \right)/2t. $$

Given expected market shares and still considering fully-coverage pollution charges, i.e., $c = \overline{c}\left( t \right)$, in the second stage, by using (4), A and B maximize own profits, respectively,

5 $$ \begin{gathered} \pi_{A}^{U} \left( {f_{A}^{U} ,f_{B}^{U} ;t,\overline{c}\left( t \right)} \right) = \left( {f_{A}^{U} - \overline{c}\left( t \right)} \right)D_{A}^{U} \left( {f_{A}^{U} ,f_{B}^{U} ;t} \right)\;{\text{and}}\; \hfill \\ \pi_{B}^{U} \left( {f_{B}^{U} ,f_{A}^{U} ;t} \right) = \left( {f_{B}^{U} - s} \right)D_{B}^{U} \left( {f_{B}^{U} ,f_{A}^{U} ;t} \right) \hfill \\ \end{gathered} $$

where $s > 0$ are the UCC service fees. Notice that, in this case, B does not pay any pollution charge. Solving the system of related first order conditions, i.e., $\partial \pi_{A}^{U} \left( \cdot \right)/\partial f_{A}^{U} = 0$ and $\frac{{\partial \pi_{B}^{U} \left( \cdot \right)}}{{\partial f_{B}^{U} }} = 0$, it yields second-stage asymmetrical equilibrium rates^6^:

6 $$ f_{A}^{U} \left( s \right) = \frac{2}{3} + \frac{s}{3}\;{\text{and}}\;f_{B}^{U} \left( {s;t} \right) = \frac{1}{3} + \frac{2s}{3} + \frac{t}{2} $$

As the competition between LSPs is in *strategic complements* (i.e., LSPs set prices of differentiated services; see Bulow et al. 1985), it is easy to check that UCC service fees raise both the freight transport rates. Interestingly, notice that switching costs (as a source of market power for LSPs) tend to raise only B’s final rates, reflecting the price-enhancing effect of *double marginalization* (e.g., Riordan 2008). In this case, yet this would harm B’s ability to gather more demand.

Going backward, in the first stage, the choice of service fees is assumed to be explicitly affected by CSR strategies adopted by the UCC management board. As said, our aim is here to model an UCC whose business model might range from a pure profit-maximizing to a full CSR-oriented behaviour, referring to all the firm’s social and environmentally friendly activities beyond its legal requirements (Kitzmueller and Shimshack 2012). Following Benabou and Tirole (2010), in the freight industry the decision by UCC managers to pursue CSR-oriented pricing schemes is inherently related to the fact that not only own final customers’ interests but also those of consumers served by other LPSs are looked after by means of pricing schemes.

As a result, in the first stage of the market game, the UCC maximizes its own wealth function:

7 $$ W^{U} \triangleq \pi^{U} + \theta \cdot CS^{U} $$

where $\pi^{U} = s \cdot D_{B}^{U} \left( {f_{B}^{U} \left( {s;t} \right),f_{A}^{U} \left( s \right)} \right)$, $CS^{U} = \mathop \smallint \limits_{0}^{{D_{A}^{U} }} \Big( {1 - tx - f_{A}^{U} } \Big)dx + \mathop \smallint \limits_{{D_{A}^{U} }}^{1} \Big( 1 - t\Big( 1 - x \Big) - f_{B}^{U}  \Big)dx$, and $0 \le \theta \le 1$ captures the degree of CSR orientation within the UCC’s pricing strategy, where $\theta =1$ corresponds to a fully CSR-oriented behaviour.^7^ Solving the first order condition, i.e., $\partial W^{U} /\partial s = 0$, we obtain equilibrium UCC fees^8^:

8 $$ s\left( {t;\theta } \right) = \frac{{3t\left( {3 - 5\theta } \right) + 2\left( {3 - \theta } \right)}}{{2\left( {6 - \theta } \right)}}. $$

Finally, by inserting Eq. (8) in Eqs. (4)–(6), we derive the post-UCC equilibrium values:

9 $$ f_{A}^{U} \left( {t;\theta } \right) = \frac{{t\left( {3 - 5\theta } \right) + 2\left( {5 - \theta } \right)}}{{2\left( {6 - \theta } \right)}}\;{\text{and}}\;f_{B}^{U} \left( {t;\theta } \right) = \frac{{t\left( {12 - 11\theta } \right) + 2\left( {4 - \theta } \right)}}{{2\left( {6 - \theta } \right)}}, $$

10 $$ D_{A}^{U} \left( {t;\theta } \right) = \frac{{t\left( {21 - 8\theta } \right) - 2}}{{4t\left( {6 - \theta } \right)}}\;{\text{and}}\;D_{B}^{U} \left( {t;\theta } \right) = \frac{{t\left( {3 + 4\theta } \right) + 2}}{{4t\left( {6 - \theta } \right)}}, $$

11 $$ \pi_{A}^{U} \left( {t;\theta } \right) = \frac{{\left[ {2 - t\left( {21 - 8\theta } \right)} \right]^{2} }}{{8t\left( {6 - \theta } \right)^{2} }}\;{\text{and}}\,\pi_{B}^{U} \left( {t;\theta } \right) = \frac{{\left[ {2 + t\left( {3 + 4\theta } \right)} \right]^{2} }}{{8t\left( {6 - \theta } \right)^{2} }}. $$

Finally, the post-UCC social welfare is:

12 $$ \begin{aligned} & SW^{U} \left( {t;\theta } \right) \triangleq \pi_{A}^{U} \left( {t;\theta } \right) + \pi_{B}^{U} \left( {t;\theta } \right) + \mathop \int \limits_{0}^{{D_{A}^{U} }} \left( {1 - tx - f_{A}^{U} } \right)dx + \mathop \int \limits_{{D_{A}^{U} }}^{1} \left( {1 - t\left( {1 - x} \right) - f_{B}^{U} } \right)dx \hfill \\ & \quad  = \frac{{20 + 12t\left( {8\theta - 3} \right) + \left( {128\theta^{2} - 240\theta + 477} \right)t^{2} }}{{16t\left( {6 - \theta } \right)^{2} }}. \hfill \\ \end{aligned} $$

### Comparison of the Two Scenarios and Main Results

To complete the analysis, in this section we look for insights coming from the interplay between the degree of CSR strategies adopted by UCCs and the horizontal differentiation in the UFT market, that we modelled by considering the intensity of costs potentially incurred by receivers from *switching* LSPs. Essentially, we are interested in establishing whether the LSPs’ incentives to outsource last-mile deliveries to UCCs might be theoretically affected by the level of CSR orientation. To do so, we compare equilibrium outcomes related to the UCC-based LSP in the pre- and post-UCC scenarios. By defining $\Delta \pi_{i} \triangleq \pi_{i}^{U} \left( {t;\theta } \right) - \pi_{i} \left( t \right)$ and $\Delta D_{i} \triangleq D_{i}^{U} \left( {t;\theta } \right) - D_{i} \left( t \right)$, for $i = A,B$, as the difference of LSPs’ profits and market shares in case of voluntary (and unilateral) decision by B to apply for UCC services, we state the following:

**Result 1** *In fully covered UFT markets, outsourcing last-mile deliveries to an UCC would let a LSP gain larger market shares and profits with respect to other competitors if the contracted UCC’s orientation towards CSR-based pricing schemes is enough large with respect to the horizontal differentiation between LSPs, i.e., only if* $\theta \ge \theta^{U} \left( t \right) \equiv \frac{9t - 2}{{6t}}$*.*

The above result can be explained as follows. By inspecting the equilibrium UCC service fee, notice that, for relatively high $\theta$, the UCC would take a stronger socially responsible stance and it is more willing to accept lower service fees in the short run.^9^ Apart from sacrificing profits, this strategy can be interpreted as a commitment device to reach long-run goals, including the ability to spread out more eco-friendly deliveries and reduce CO_2_ emissions to the benefit of the whole society. Actually, this pricing behaviour by the UCC would also fairly strengthen the contracted LSP’s market position and provide her with larger and stable market shares. Formally, it happens that $\Delta D_{B} > 0$ for $\theta \ge \theta^{U} \left( t \right)$. Since the UFT market is assumed to be fully covered, this also implies that $\Delta D_{A} < 0$. But, in that parameters’ region, is the UCC-based LSP expected to gain also larger profits with respect to the pre-merger scenario? Alongside to increasing market shares, it is rather easy to prove that, for $\theta \ge \theta^{U} \left( t \right)$, also $\Delta \pi_{B} > 0$ (and $\Delta \pi_{A} < 0$).^10^

**Fig. 3:**
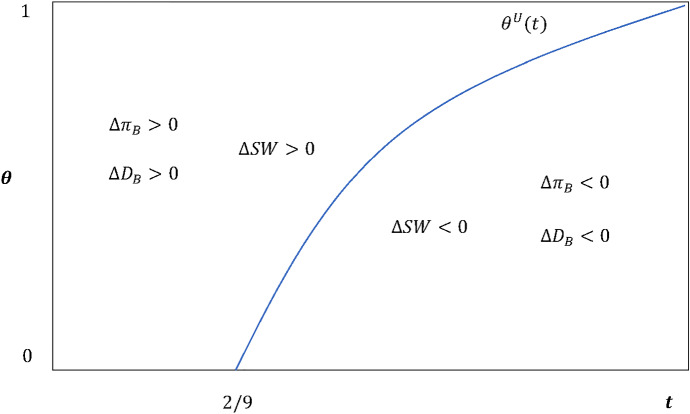
Contracted LSP’s market shares and profits, and social welfare. The effect of CSR-based pricing schemes ($\theta$) in function of the horizontal differentiation between LSPs (*t*)

As for the role played by the receivers’ preference about LSPs, as depicted in Fig. 3, for a relatively weak horizontal differentiation (i.e., $t < 2/9$), both the B’s market-shares and profits are dominant over A for any level of CSR orientation. In this case, by recalling the Remark 1, notice that, for smaller switching costs (low *t*), the full-coverage pollution charges would be relatively higher. In turn, this implies that environmental policies (in the form of higher costs) might let UCC-based LSPs’ services be more affordable in itself, despite the presence of the *double marginalization* effect. For larger switching costs (high *t*), instead, the impact of pollution charges would vanish, therefore larger shares and profits for B are ensured only if the CSR behaviour by the UCC is strong enough.

Therefore, from a social and environmental welfare perspective, pollution charges to enter LEZs and the intensity of CSR-based pricing schemes by UCCs might operate as weak substitutes to help reducing the number of polluting deliveries in the last mile. Furthermore, since for $\theta \ge \theta^{U} \left( t \right)$ it is checked that $\Delta SW \triangleq SW^{U} \left( {t;\theta } \right) - SW\left( t \right) > 0$, we can also conclude that the potential rise of a UCC-based dominant LSPs in the market would not actually harm the total surplus (profits plus consumer welfare), as stressed in the following^11^:

**Result 2** *In order to enhance the social welfare and favour the diffusion of eco-friendly deliveries, environmental policies (e.g., pollution charges) and levels of CSR orientation by UCCs are weak substitutes.*

## Concluding Remarks

Due to the increasing engagement by oligopolistic firms in CSR activities, the academic and political debate on the reasons inducing companies to engage in socially concerned actions has become prominent. Even in UFT markets, this topic assumed a relevant importance, especially for the role that LSPs might have in the development of sustainable supply chains. Together with environmental policies designed by local institutions to reduce CO_2_ emissions in freight transportation, in this paper we considered the deployment of UCCs, that is, logistics platforms devoted to last mile consignments. In the last years, successful examples of UCCs have displayed business models integrating CSR behaviours, in the form of pricing schemes where the whole consumer welfare (i.e., all the receivers in an urban area) is taken into account. 

By using a Hotelling-like duopolistic framework and considering the existence of pollution charges to regulate access in the city centre, as it happens in reality in various urban areas, we study the effect of the introduction of an UCC into an UFT market. We focus our attention on the interplay between the degree of CSR-based pricing schemes adopted by the UCC and the intensity of horizontal differentiation between LSPs (measured by switching costs). Though simple, our model suggests interesting insights about the ability by CSR strategies to expand the market shares of LSPs outsourcing last mile deliveries to UCCs and to enhance the diffusion of eco-friendly deliveries. Notably, these implications rely on competition characteristics of the UFT markets, so their scope would apply for different levels of contestability in last mile deliveries. Actually, when the competition among LSPs is strong (i.e., switching costs are relatively weak), we suggest that UFT markets are more contestable, thus the design of environmental policies, in the form of enough high pollution charges, might give the LSPs sufficient incentives to apply for UCC-based deliveries, as they would be performed by free-of-charge green vehicles. In a sense, we argue that pollution charges alone are able to give UCC-based LSPs a competitive advantage over other providers. This is an important insight, as it states that local public policies might impact on the diffusion of greener consignments regardless CSR-oriented pricing schemes. However, this result applies only when the competition among LSPs is enough fierce. By contrast, when the competition is weak (e.g., due to high switching costs between providers), LSPs have a relevant market power and they would be incentivized to contract UCCs only if these platforms adopt CSR-based pricing schemes, oriented to the maximization of whole society’s interests and final customers’ utility. In this case (that is far more common in modern UFT markets), the implementation of UCCs could be successful in sustainability terms and attract LSPs’ attention only when the consideration of CSR arguments is properly taken into account by means of pricing schemes oriented to the whole set of urban customers, i.e., the entire consumers’ society.

Despite the obtained results are of notable relevance in the context of CSR strategies in the city logistics, the present work still has some limitations, which however do not negatively affect the overall results obtained. First, as we moved from Hotelling-style theoretical foundations, actually our analysis gives a stylized representation of the UFT competition. Some assumptions on costs and demand-related features (e.g., the *per-customer* demand could be alternatively modelled as a continuously decreasing function of the freight transport rates) might overlook still key operational elements, such as the scale effects of load consolidation. In this sense, future next steps of the research will aim at empirically validating the model, mainly by collecting data from case studies of successful UCCs and by conducting interviews with LSPs, retailers, municipal delegates and other stakeholders involved. Hence, we would have a more detailed landscape of UCCs’ business models integrating CSR strategies, and the ability to test or calibrate the validity of our promising theoretical conclusions.

Despite these limitations and being a primer and theoretical research, the results of this work could be of interest both for policymakers and LSPs and UCCs’ managers. As regards the first ones, the paper reinforces the idea that UCC could still be an effective solution to contain the growing negative externalities of freight transport in the era of e-commerce, but it needs to be reinforced by complementary public policies based on pollution charges for the LSPs not adopting the greener UCC scheme. From a managerial perspective, our analysis shows that CSR-based pricing schemes adopted by consolidation platforms might enhance the demand coming from LSPs, aiming to decrease their carbon footprint, giving their contribution to the challenge of sustainability and, by this way, to reinforce their customer loyalty scheme and, as a consequence, their market position.

## Appendix

### A.1 Proofs of Result 1

In order to determine the expected gains from outsourcing UCC-based services, we start from potential larger market shares for B. Formally, $\Delta D_{B} = \frac{{t\left( {3 + 4\theta } \right) + 2}}{{4t\left( {6 - \theta } \right)}} - \frac{1}{2} = \frac{{2 - 3t\left( {3 - 2\theta } \right)}}{{4t\left( {6 - \theta } \right)}}$, which is equal to zero for $\theta = \theta^{U} \left( t \right)$. Since $\frac{{\partial \Delta D_{B} }}{\partial \theta } = \frac{2 + 27t}{{4t\left( {6 - \theta } \right)^{2} }} > 0$, it is easy to prove that, for $\theta > \theta^{U} \left( t \right)$, B would gather more demand than the pre-UCC scenario. As, in our duopolistic setting, changes in market shares are zero-sum figures, then, in that region, $\Delta D_{A} < 0$. Regarding to expected profit gains, higher market shares imply larger profits as well, i.e., $\Delta \pi_{B} = \frac{{\left[ {2 + t\left( {3 + 4\theta } \right)} \right]^{2} }}{{8t\left( {6 - \theta } \right)^{2} }} - \frac{t}{2} = \frac{{4\left[ {1 + t\left( {3 + 4\theta } \right)} \right] + 3t^{2} \left( {4\theta^{2} + 24\theta + 45} \right)}}{{8t\left( {6 - \theta } \right)^{2} }} > 0$ for $\theta > \theta^{U} \left( t \right)$. Still, this means that the non-UCC LSP A, for that region of parameters, would gain lower profits ($\Delta \pi_{A} < 0$).

### A.2 Proofs of Result 2

The difference between post- and pre-UCC social welfare is: $\Delta SW = \frac{{20 + 12t\left( {8\theta - 3} \right) + 27t^{2} \left( {4\theta^{2} - 9} \right)}}{{16t\left( {6 - \theta } \right)^{2} }}$. As B would contract the UCC only for $\theta \ge \theta^{U} \left( t \right) \equiv \frac{9t - 2}{{6t}}$ to gain larger profits, it is easy to prove that, for those levels of CSR orientation, $\Delta SW \ge 0$.

## Footnotes

2 Since incentives are vital to support UCCs, the charges would depend on several aspects, such as the size of the vehicle, engines used, and access time (Björklund et al. 2017). From a normative point of view, this parameter can be also interpreted as a proxy for sharing polluting vans used in the industry. Higher levels of *c* would mean that, on average, LSPs use highly polluting vehicles (e.g., diesel vehicles Euro 0, 1, 2, with PM_10_ emission factors from 50 to more than 100 mg/km).

3 Second-order conditions for local maxima are fulfilled, as $\partial^{2} \pi_{A} \left( {f_{A} ,f_{B} ;t,c} \right)/\partial f_{A}^{2} = \partial^{2} \pi_{B} \left( {f_{B} ,f_{A} ;t,c} \right)/\partial f_{B}^{2} = - 1/t < 0$.

4 That is, by imposing $V_{A} \left( {x = D_{A} \left( {f_{A} ,f_{B} ;t} \right)|_{{f_{A} ,f_{B} }} } \right) = 1 - \frac{3}{2}t - c \ge 0$.

5 Without loss of significance, we rule out deliveries outside the city centre performed by A and B.

6 Second-order conditions for local maxima are fulfilled, as ${\partial }^{2}{\pi }_{i}^{U}\left(\cdot \right)/\partial {{f}_{i}^{U}}^{2}=-1/t<0$ for $i=A,B$.

7 For sake of simplicity, let the UCC operating costs be nihil, meaning that revenues are maximized.

8 Since $\frac{{\partial^{2} W^{U} }}{{\partial s^{2} }} = - \frac{6 - \theta }{{18t}} < 0$, we check that second-order conditions are satisfied.

9 Formally, $$\frac{{\partial s\left( {t;\theta } \right)}}{\partial \theta } = - 3\left( {2 + 27t} \right)/2\left( {6 - \theta } \right)^{2} < 0$$.

10 Proofs of Result 1 are in the “Appendix A.1”.

11 Proofs of Result 2 are in the “Appendix A.2”.
